# High procalcitonin level is related to blood stream infections, gram-negative pathogens, and ICU admission in infections of adult febrile cancer patients

**DOI:** 10.1186/s43046-025-00283-1

**Published:** 2025-05-10

**Authors:** Hadir Ahmed El-Mahallawy, Nourhan Ahmed Zakaria, Afaf Mohamed El Banna, Mohamed Ghareeb

**Affiliations:** 1 National Cancer Institute Cairo University, Cairo, Egypt; 2 Kasr Al Aini Cairo University, Cairo, Egypt

**Keywords:** Procalcitonin (PCT), C-reactive protein (CRP), Febrile cancer patients, Blood stream infection (BSI), Gram-negative bacteremia

## Abstract

**Background:**

Blood stream infection (BSI) represent a life-threatening condition. Thus, we aimed to investigate the role of procalcitonin (PCT) and C-reactive protein (CRP) tests in adult febrile patients with BSI and other clinical infections in hospitalized cancer cases.

**Methods:**

Blood culture (BC) testing was performed using BACTEC 9120. Identification and antibiotic susceptibility were done by Vitek 2®. Multiplex PCR for the detection of carbapenemases genes produced by Enterobacteriaceae was carried out including *KPC*, *NDM*, *IMP*, *VIM*, and *Oxa-48* genes. Measurement of CRP was done via particle-enhanced immunoturbidimetric assay using Cobas C6000 autoanalyzer. PCT level was measured using the electrochemiluminescence immunoassay.

**Results:**

Out of 101 febrile hospitalized adult cancer cases with clinical infection, 50 had positive BC, and 51 were positive for other infections (27 localized bacterial and 24 viral infections) with a negative BC. At a PCT cut-off value of 0.5 ng/mL, PCT median values were significantly higher in BSI patients than those with other infections (*p* = 0.004), specifically with gram-negative BSIs (*p* = 0.007). Higher PCT values were significantly related to ICU admission and poor response to therapy, *p* = 0.004 and 0.002, respectively. The difference in CRP values between patients with BSI and other febrile cases was not statistically significant, *p* = 0.922.

**Conclusion:**

Higher PCT values were significantly related to blood stream infections, gram-negative pathogens, ICU admission, and poor response to therapy. Procalcitonin could be used to assign severity of infection and monitor response to antimicrobial therapy in high-risk patients, thus reducing days of antibiotics days.

## Introduction

Infections are still one of the most common complications of cancer management. Infection in cancer patients is accompanied with increased morbidity and mortality as well as delayed treatment regimens, prolonged hospitalization, and increased financial burden of health care [1]. Blood stream infections (BSIs) are the most life-threatening conditions among other causes of infections [2]. This is especially dangerous in the era of antimicrobial resistance (AMR) [2, 3]. Neutropenic sepsis when suspected is considered a medical emergency, and immediate empiric antibiotic therapy should be offered [4]. A good marker is supposed to help the early diagnosis of infection in general and sepsis in particular to serve a timely appropriate antimicrobial therapy, at the same time avoiding unnecessary prolonged use of antimicrobial agents. This strategy could help to avoid adding to the burden of AMR.

Procalcitonin (PCT) is a hormokine and is the precursor of calcitonin. It is widely produced by many organs as well as macrophages. Among its major advantages as a marker of infection are it is produced within few hours of an inflammatory process, peaks within 24 h, and have a half-life of 22 to 35 h [5]. C-reactive protein as the most widely used inflammatory marker is an acute-phase protein with a longer half-life.

Procalcitonin (PCT) is considered a breakthrough in sepsis management. Its level increases in bacterial infections and decreases if treatment is successful. Measuring PCT has been used to guide antibiotic therapy in critically ill patients. This conclusion was based on several randomized controlled studies in various clinical severity demonstrating the efficacy of PCT-guided antibiotic decision-making. Another benefit of PCT has been the prediction of outcome in postoperative critically ill patients with severe sepsis [6]. The use of PCT in the management of sepsis has been confirmed by many studies with the advantage of rapid diagnosis and risk assessment of severity of infection [7]. However, PCT levels might not be helpful in localized infections and rarely increases in response to viral infections [8]. Fever may be the only sign of infection in cancer patients, and they might lack the typical presentation of infection. It is difficult in the early days of infection to differentiate between BSI or other causes of infection. Thus, markers of infection are needed to confirm the probability of infection and assign, if possible, more severe infections to aid in the antibiotic decision-making. In the present study, we aimed to investigate PCT and CRP levels in BSI versus febrile patients with clinical evidence of infection in hospitalized cancer cases and their relation to risky infections like BSI, type of pathogen, and response to therapy.

## Patients and methods

*Patient population* were hospitalized adult cancer patients who developed fever and clinical evidence of infection while receiving therapy at the National Cancer Institute (NCI), Cairo University. They were either febrile neutropenic or cancer patients undergoing surgery. In the period from September 2020 until October 2021, 2111 febrile patients were subjected to blood culture testing. Other cultures from suspected infectious sites were done. Viral studies were performed whenever viral infections were clinically suspected.

The inclusion criteria were adult cases with clinical evidence of infection and fever ≥ 48 h. Patients with evidence of infection were monitored, and those with a positive lab result either bacterial or viral were included. Exclusion criteria were patients younger than 18 years, fever less than 48 h, stem cell transplant cases, and pregnancy. Clinical data of the studied cases were recorded including age, diagnosis, fever grade, and fever duration. Cause of documented infections, antibiotic received, response to therapy, ICU admission, episode duration, and outcome within 1-month duration of the episode were documented. Hematological laboratory data were recorded including total leucocytic count (TLC), absolute neutrophil count (ANC), absolute monocytic count (AMC), and absolute lymphocyte count (ALC). Ethical approval was obtained from the Research Ethical Committee of Faculty of Medicine, Cairo University.

A control group comprised of 25 hospitalized cancer patients with the same circumstances as study group, but without fever, clinical or microbiological evidence of infection were included.

*Microbiology febrile* neutropenic patients are routinely subjected to blood culture testing according to NCI guidelines by using BACTEC 9120, New Jersey, USA. Identification and antibiotic susceptibility testing of positive cultures were done by Vitek 2, bioMerieux, Marcy, l’Etoile, France. In patients showing localized infections, cultures were done from sites of infection according to CLSI standards. Multiplex PCR for the detection of carbapenemases genes produced by Enterobacteriaceae was carried out including *KPC*, *NDM*, *IMP*, *VIM*, and *Oxa-48* genes [9].

Viral infections are laboratory investigated when clinically suspected, namely severe acute respiratory syndrome coronavirus-2 (SARS-CoV-2), hepatitis C virus (HCV), hepatitis B virus (HBV), and human immunodeficiency virus (HIV). Human herpes virus is clinically diagnosed.

### Markers of inflammation

Measurement of CRP was done using Cobas C6000 autoanalyzer, Roche, Manheim, Germany, via particle-enhanced immunoturbidimetric assay. Procalcitonin level measurement was done using Roche Elecsys BRAHMS PCT immunoassay Cobas Eu 411 Roche, Manheim, Germany, via the electrochemiluminescence immunoassay; ECLIA technique. The auto-analyzer automatically calculated the analyte concentration of each sample in ng/mL [10].

*Response to therapy* was evaluated as complete response with no fever, negative culture, and resolution of clinical manifestations of infection within 10 days or less to antimicrobial therapy. Partial response was reported if the patient responded in more than 10 days and within 14 days. Failure of response was recorded if manifestations of infection persisted for > 14 days or unfavorable outcome. Crude mortality is mortality recorded within 1 month of the infectious episode.

### Statistical methods

Statistical analysis was done using IBM SPSS® version 26 (IBM Corp., Armonk, NY, USA). Numerical data were expressed as mean and standard deviation or median and range as appropriate. Qualitative data were expressed as frequency and percentage. Pearson’s chi-square test or Fisher’s exact test was used to examine the relation between qualitative variables. Comparison of quantitative data between the two groups was done using Student’s *t*-test for normally distributed data or Mann–Whitney test (nonparametric *t*-test) for not normally distributed data. Kappa test was used to evaluate the agreement between the results of two inflammatory markers CRP and PCT. All tests were two-tailed. A *p*-value < 0.05 was considered significant.

## Results

This is a case–control study. A total of 101 adult patients (> 18 years) presenting with 48 h of fever and clinical evidence of infection in medical and surgical wards at the National Cancer Institute were included in the study in the period from September 2020 to October 2021. Of these patients, 50 had positive blood culture, and 51 had negative blood culture results.

### Epidemiological features of patients

Among the positive blood culture group, age ranged from 20 to 73 years with a median of 47 years, 52% (*n* = 26) of patients < 50 years. They were 22 (44%) males and 28 (56%) females. Among the negative blood culture group, age ranged from 19 to 70 years with a median of 45 years, 60.8% (*n* = 31) of patients < 50 years. They were 28 (54.9%) males and 23 (*45.1%*) females. The afebrile control group was 10 males and 15 females with a median age of 45 years (range 19 to 69). Regarding patients’ diagnoses, hematological and solid malignancies were represented in 51.5% (*n* = 52) and 48.5% (*n* = 49) of patients, respectively. Among the 101 cases, 52 (51.5%) were in medical wards and 49 (48.5%) in surgical wards. Ten cases of the control group (40%) were in medical wards and 15 (60%) in surgical wards.

### Clinical characteristics of patients

Neutropenia < 500 × 10)9(/L, monocytopenia < 700 × 10)9(/L, and lymphopenia less than 1000 × 10)9(/L were recorded in 14/50 (28%), 41/50 (82%), and 39/50 (78%) of BSI cases and 17/51 (33%), 38/51 (75%), and 33/51 (65%) of those with a negative BSI, respectively, with a *p*-value 0.561, 0.362, and 0.226, respectively. ICU admission in positive BSI group was 38 (*76.0%*) and in the negative BSI was 21 (41.2%), with a statistically significant difference, *p*-value < 0.001.

### Microbiology

**Fig. 1:**
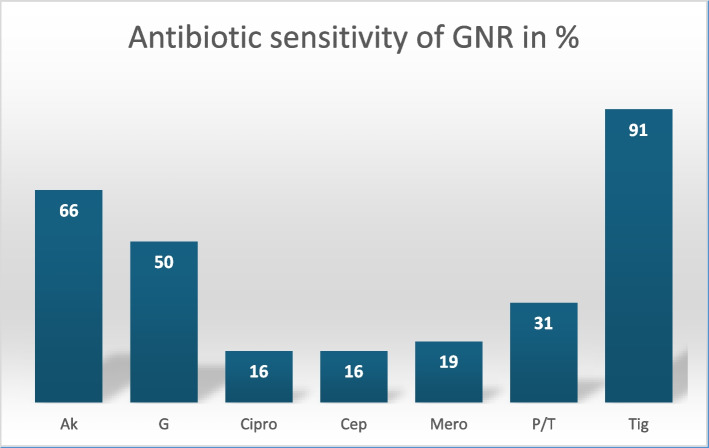
Antibiotic sensitivity of 36 g-negative pathogens isolated from blood cultures of 50 patients with blood stream infections. GNR, gram-negative rods. Ak, amikacin; G, gentamicin; Cipro, ciprofloxacin; Cep, cefepime; Mero, meropenem. P/T, piperacillin-tazobactam; Tig, tikacillin

In the group of patients with positive BSI, 64% of the organisms isolated were gram-negative organisms and 36% gram-positive bacteria. The different organisms isolated are shown in Table 1. Of the gram-negative organisms, 6/32 (18.7%), 26/32 (81.3%), and 24/32 (75%) were extended spectrum B-lactamase (ESBL), multidrug resistance (MDR), and carbapenem-resistant Enterobacteriaceae (CRE), respectively. Antibiotic sensitivity pattern of the gram-negative pathogens is summarized in Fig. 1. Carbapenemases genes produced by Enterobacteriaceae including *KPC*, *NDM*, *IMP*, *VIM*, and *Oxa-48* revealed 13 (40.6%) positive results among 32 g-negative organisms isolated from positive blood culture. Of the carbapenemases genes, oxa-48 was detected in 11 cases, 8 of *Klebsiella pneumoniae*, and 3 *Escherichia coli*. The NDM gene was detected in five cases.

**Table 1:** Different organisms isolated from blood cultures of 50 consecutive patients with blood stream infections among febrile cancer patients

Gram-positive bacteria *N* = 18 (36%)		Gram-negative bacteria *N* = 32 (64%)	
*S. aureus*	3	Enterobacteriaceae	25
CONS	11	o *K. pneumonia*	18
		o *Klebsiella oxytoca*	1
		o *E. coli*	5
		o *Proteus*	1
*Streptococcus*	4	Non-fermenters	7
		o *A. baumannii*	4
		• *P. aeruginosa*	3

Within the group with a negative BSI, 27/51 (53%) showed positive cultures from localized sites of infection. Gram-negative organisms isolated in the BSI-negative group (*n* = 15) were *K. pneumoniae*, *E. coli*, *Acinetobacter baumannii*, and *Pseudomonas aeruginosa* in seven, four, three, and one patients, respectively, whereas gram-positive organisms isolated (*n* = 12) were *Staphylococcus aureus*, streptococcal species, and CoNS in five, four, and two cases, respectively, with a single case of *Candida* species.

Viral infections were documented in 24/51 (47%) patients with a negative blood culture result. These were SARS-CoV-2, HCV, human herpes virus, HIV, and dual infection HCV + HBV in 14, 5, 3, 1, and 1, respectively.

### Clinical response

Patients received antibiotic therapy according to hospital guidelines. Response to antimicrobial therapy was successful within 10 days, partial recovery, or poor recovery in 26 (52%), 14 (28%), and 10 (20%) in the BSI cases and in 34 (67%), 9 (17%), and 8 (16%), respectively, *p*-value 0.306. Antibiotic shift was needed in 38 (76%) of the BSI-positive group and in 24 (47%) BSI-negative group, *p*-value 0.003. Colistin was added in 22 (44%) of the BSI patients and in 14 (27%) of those with no BSI. Crude mortality was 38% (*n* = 19) in patients with BSIs, while it was 18% (*n* = 9) in patients with other infections and negative for BSI. Higher mortality was significantly related to BSI, *p*-value 0.022.

### Markers of inflammation

The median levels of PCT in the control group, BSI group of patients, and the group with negative blood culture were 0.14 (0.02–0.39) ng/mL, 4.44 (0.07–184) ng/mL, and 0.70 (0.02–210.0) ng/mL, respectively. The difference in PCT values between patients with BSI and control group as well as between cases with no BSI and control group was statistically significant with a *p*-value *0.02* and *0.045*, respectively. The median levels of CRP in the BSI group of patients and the group with negative blood culture were 148.1 (6.0–411.0) ng/mL and 139.0 (1.1–348.0) ng/mL, respectively. The difference in CRP values between patients with BSI and cases with no BSI group was not statistically significant with a *p*-value *0.922*.

A PCT level higher than 0.5 was detected in 41/50 (82%) BSI cases and in 27/51 (53%) of cases with a negative blood culture result. The difference between both groups as regards PCT cutoff was statistically significant (*p*-value 0.011). The relation between inflammatory markers with clinical findings and clinical response to antimicrobial therapy in cancer patients was included in the study, and microbiology results are summarized in Tables 2 and 3, respectively.

**Table 2:** The serum levels of PCT and CRP in relation to clinical findings and clinical response to antimicrobial therapy in 101 adult febrile cancer patients with infection

Clinical findings	PCT	*p*-value	CRP	*p*-value
**ICU admission**		0.004		0.230
**Yes, *n* = 59**	2.93 (0.04–184.4)		174.8 (6.0–411)	
**No, *n* = 42**	0.60 (0.02–210)		134.4 (1.1–313)	
**Serum bilirubin**		< 0.001		0.026
> **1.2 mg/dL**	4.5 (0.2–91.0)		208.0 (11.7–411.0)	
≤ **1.2 mg/dL**	0.6 (0.02–210.0)		126.4 (1.1–341.8)	
**Duration of fever**		0.339		0.173
< **7 days**	1.74 (0.02–210)		140.7 (1.1–411)	
**7–10 days**	0.57 (0.03–48.5)		123.2 (6.0–297)	
> **10 days**	3.29 (0.2–91.0)		146.1 (56.9–311.8)	
**Antibiotic shift**		0.914		0.889
**Yes, *n* = 62**	1.6 (.04–91.0)		145.4 (6.0–348)	
**No, *n* = 39**	1.7 (0.02–210)		138.7 (1.1–411.0)	
**Response**		0.002		0.215
**Complete**	1.24 (0.02–210)		134.4 (1.1–341.8)	
**Partial**	0.55 (0.07–39.4)		139.9 (6.0–411.0)	
**Poor**	8.95 (0.1–154.9)		194.6 (46.2–348.0)	

*p*-values less than 0.05 are considered significant

**Table 3:** The serum levels of PCT and CRP in relation to microbiology results in 101 adult cancer patients presenting with fever and infection

Microbiology results	PCT^a^	*p*-value	CRP^b^	*p*-value
Blood culture		0.004		0.922
Yes, *n* = 50	4.4 (0.1–184)		148 (6.0–411)	
No, *n* = 51	0.7 (0.0–210)		139 (1.1–348)	
Blood culture		0.007 GN vs NG 0.005 GP vs NG 0.609 GP vs GN 0.682		0.965
GNR, *n* = 31	4.7 (0.1–184.4)		139 (13.6–411)	
GPC, *n* = 19	1.9 (0.1–22.2)		163 (6.0–297)	
NG, *n* = 51	0.7 (0.0–210)		139 (1.1–348)	
CRE		0.115		0.678
Yes, *n* = 22	3.6 (0.1–184.4)		132.6 (13.6–411.0)	
No, *n* = 79	1.1 (0.0–210)		142.6 (1.1–348.0)	
Viral infection		0.394		0.920
Yes, *n* = 24	0.7 (0.1–48.5)		151.5 (11.7–341.8)	
No, *n* = 77	1.76 (0.0–210.0)		139.5 (1.1–411.0)	

^a^Values of PCT are in ng/mL

^b^Values of CRP are in mg/L. *P*-values less than 0.05 are considered significant. *NG*, no growth in blood culture test; *GNR*, gram-negative rods; *GPC*, gram-positive cocci; *CRE*, carbapenem-resistant Enterobacteriaceae

Out of the 32 GNR pathogens isolated from positive blood cultures, 5 had a PCT level below 0.5, 4 of whom showed an elevated PCT above 0.5 in the follow-up sample.

#### ROC curve

**Fig. 2:**
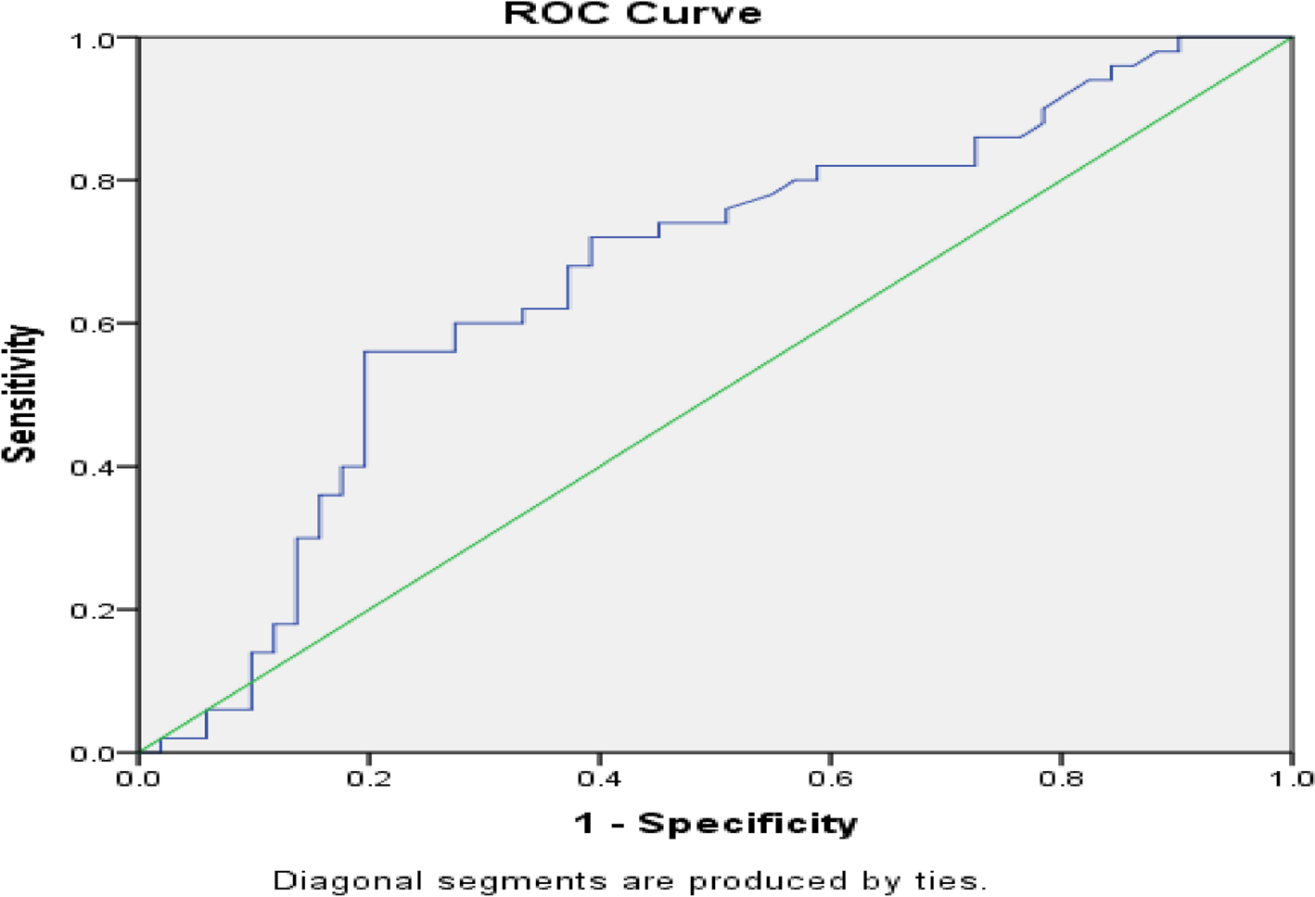
ROC curve of PCT levels in patients with blood stream infections in relation to those with clinical evidence of infection and negative blood culture results. *AUC* 0.667 (95% *CI*: 0.560–0.775)

A PCT cut-off value of 0.5 ng/mL to discriminate BSI and febrile clinically infected cases with no BSI yielded 82% sensitivity, 41% specificity, 58% positive predictive value (PPV), and 70% negative predictive value (NPV). Area under the curve for PCT serum levels was 0.667 with 95% confidence interval 56–0.78% (Fig. 2). The sensitivity, specificity, PPV, NPV, and accuracy of different PCT cut-off values are summarized in Table 4.

**Table 4:** Sensitivity, specificity, PPV, NPV, and accuracy of different cut-off levels of serum PCT in 50 patients with blood stream infection in comparison to 51 patients with evidence of clinical infection included in the study

PCT cutoff	Sensitivity	Specificity	PPV	NPV	Accuracy
≥ 0.25 ng/mL	88%	22%	52%	65%	55%
≥ 0.5 ng/mL	82%	41%	58%	70%	61%
≥ 1.1 ng/mL	72%	59%	63%	68%	65%
≥ 1.4 ng/mL	68%	61%	63%	66%	64%

## Discussion

Infections are serious complications of chemotherapy-induced neutropenia in patients with hematologic and solid organ malignancies [11]. Bacterial BSIs account for the etiologic cause of approximately 20 to 30% of all febrile neutropenic episodes in adult patients with malignancy [12]. In the era of the rapidly accelerating risk of antimicrobial resistance, it is essential to better understand the epidemiological changes in infections in cancer patients for appropriate and timely antimicrobial therapy. Inflammatory markers can be useful for clinical decisions in the initial assessment. Procalcitonin has been reported as a reliable and specific biomarker of infections especially bacterial infections [2].

Thus, PCT and CRP were investigated in relation to clinical infection in hospitalized cancer patients at NCI. Out of 2111 febrile hospitalized adult cancer cases, a total of 101 adult patients presenting with 48 h of fever and clinical evidence of infection in medical and surgical wards at NCI were included in the study in the period from September 2020 to October 2021. Of these patients, 50 had positive blood culture, and 51 had negative blood culture results with other causes of infection.

PCT median values were significantly higher in BSI patients (*p* = 0.004), specifically with gram-negative BSIs (*p* = 0.007). Similar results were reported in previous studies demonstrating statistically significant higher levels of PCT values among patients with positive blood culture compared to those with negative results [13]. It was concluded that PCT level could be used as a predictor of bacteremia and sepsis in febrile cancer patients. A PCT level of > 0.25 ng/mL was a predictive factor of positive BSI in 90/260 febrile cancer patients, versus 26/277 of those < 0.25 ng/mL (*p* < 0.001) [14]. Higher PCT values were recorded in cancer patients with gram-negative BSIs compared to GPC BSI: 2.18 and 0.41 ng/mL, respectively [14]. In febrile pediatric cancer patients, cases with BSI demonstrated significantly higher PCT levels than other causes of fever in a total of 3118 episodes (*p* < 0.001). In the latter study, a PCT level of 0.78 ng/mL predicted septic shock in the area under the curve of receiver operating characteristic (ROC) curve [7].

A PCT level higher than 0.5 ng/mL was detected in 41/50 (82%) BSI cases and in 27 (53%) of 51 cases with bacterial or viral documented infection and a negative blood culture result in the present study. The difference between both groups as regards PCT cutoff was statistically significant (*p* = 0.011). Comparing PCT levels of patients with BSI to hospitalized afebrile cases would yield a 100% sensitivity and specificity. But, as the situation in practice is a febrile patient with suspected infection versus other causes of inflammation; thus, markers are important. This explains the reason that PCT has a good sensitivity to detect BSI but with a poor specificity when compared to other causes of fever. The reason behind this was that within the group with a negative BSI, 27/51 (53%) showed positive bacterial cultures from localized sites of infection. Thus, though PCT was significantly higher in the group with a positive blood culture result in the present study, still at 0.5 cut-off level it is associated with microbiological documented infections.

In adult cancer patients with solid tumors, PCT levels were significantly higher among cases with clinical and microbiological documented infections, when compared to noninfectious causes of fever with a 0.52 ng/mL cut-off level [15]. In their study, Vassalo and his colleagues (2021) concluded that PCT test could discriminate infection from cancer-associated fever with a sensitivity, specificity, PPV, and NPV of 75%, 55%, 77%, and 52%, respectively. It is evident from the findings of the current study that PCT at a cutoff of 0.5 could not be used as a marker of sepsis in febrile cancer patients; still, specificity increases with higher levels of PCT. It was recently concluded that when increasing the cut-off levels of PCT, the test specificity increased while its sensitivity decreased [16]. Our findings supported the latter conclusion, as at a cutoff 1.1 the specificity of PCT to indicate BSI increased to 59%. At a cutoff of ≥ 1.1 ng/mL, the sensitivity, specificity, PPV, NPV, and accuracy of PCT were 72%, 59%, 63%, 68%, and 65%, respectively. Further studies are recommended to investigate if higher PCT cutoff could indicate BSI.

Distinguishing causes of fever in a cancer patient is a difficult task as they are manifesting an inflammatory condition due to cancer itself, metastasis, after undergoing surgery for tumor removal, besides variable causes of infection. Thus, the low specificity of PCT in febrile cancer patients could be explained that these patients are a heterogeneous population with a wide spectrum of expected infections. Viral infections, mainly COVID-19, were not associated with elevated PCT in the current study, as was demonstrated in previous studies [8]. This could possibly be due to that viral infections activate the secretion of *α*-interferon which operates a different pathway than through PCT synthesis.

It is evident that GNR constituted the most common pathogens causing BSI (64%) as well as localized infections (56%) in the present study. Among GNR isolates, *K. pneumoniae* was the most frequently isolated bacteria constituting 60% of GNR pathogens. The antibiotic susceptibility pattern of the GNR pathogens isolated from blood cultures of our patients with BSI showed a predominance of MDR of 81% with 75% CRE. This was similar to the results of a previous study on febrile cancer patients at the same institution reporting high rates of MDR among GNR isolates [9]. Recent data from Egypt confirmed a high predominance of MDR among different governmental and university hospitals [17]. Increasing prevalence of MDR is a major problem worldwide, but this could be more critical in our high-risk patients.

In the current study, PCT values were significantly related to ICU admission, bilirubin levels, and response to therapy: *p*-value 0.004, < 0.0001, and 0.002, respectively. Patients with poor response demonstrated higher PCT levels than those with complete or partial response to antimicrobial therapy. These findings suggest that PCT test could be used to assign cases with sepsis, to guide antibiotic therapy regimens, and to be used as a diagnostic stewardship tool.

The diagnostic accuracy of CRP was lower than PCT in predicting bacteremia in the present study, with a specificity of 20% at a cutoff of 65 mg/L for CRP versus specificity of 41% for PCT. The difference in CRP values between patients with BSI and those with no BSI was not statistically significant with a *p*-value 0.922. This could be due to that all patients included in our study were documenting possible causes of inflammation in addition to clinical evidence of infection. Similar findings were reported in previous studies [18, 19]. It was concluded that PCT levels, not CRP, were significantly higher in gram-negative bacteremias than other causes of nonbacterial infections [18]. In the current study, CRP levels were significantly related to diagnosis (*p*-value 0.015) and to ICU admission suggesting a wider inflammatory role for CRP in cancer patients. This could hinder its role in guiding antibiotic therapy in febrile neutropenic cases.

### Limitations of the study

Though present study aimed to focus on the real scenario of infection in cancer patients, it was faced with the cross-link between BSI and other causes of bacterial localized infections. Cancer patients are a heterogenous population with multiple causes of inflammation and infections. Thus, cases with ≥ 48-h fever were included in the study. The major limitation of the study was the failure to randomize cases during patient selection into different groups. But this could be a difficult task with the delay in lab results to 3–5 days in most of the cases.

In conclusion, PCT levels were significantly higher in febrile cancer cases with clinical evidence of infection, when compared to afebrile hospitalized cancer cases. It was not elevated in cases with viral infections, mainly COVID-19, strengthening its role in detecting bacterial infections. At a cutoff of 0.5 ng/mL, PCT levels were more associated with blood stream infections. Increasing the cutoff more than 0.5 ng/mL, increased the specificity of PCT to indicate BSI. PCT values were significantly related to ICU admission, bilirubin levels, and response to therapy. This points to its potential beneficial role in the follow-up of the response to antimicrobial therapy. More specifically, PCT could be used to reduce antibiotic days in febrile cancer patients. Further studies are recommended to investigate if PCT use could reduce the duration of inappropriate antibiotics aiming for shorter periods of antimicrobial therapy and reducing costs. This could be especially important with the rising rates of AMR.

## Data Availability

No datasets were generated or analysed during the current study.
